# Diversity of burial rates in convergent settings decreased as Earth aged

**DOI:** 10.1038/srep26359

**Published:** 2016-05-24

**Authors:** Gautier Nicoli, Jean-François Moyen, Gary Stevens

**Affiliations:** 1 Center for Crustal Petrology, Earth Sciences Department, University of Stellenbosch, Private Bag X1, 7602 Matieland, South Africa; 2 Univ. Lyon, UJM-Saint-Etienne, UBP, CNRS, IRD, Laboratoire Magmas et Volcans UMR 6524, F-42023 Saint Etienne, France

## Abstract

The evolution and the growth of the continental crust is inextricably linked to the evolution of Earth’s geodynamic processes. The detrital zircon record within the continental crust, as well as the isotopic composition of this crust, indicates that the amount of juvenile felsic material decreased with time and that in geologically recent times, the generation of new crust is balanced by recycling of the crust back into the mantle within subduction zones. However it cannot always have been so; yet the nature of the crust and the processes of crustal reworking in the Precambrian Earth are not well constrained. Here we use both detrital zircon ages and metamorphic pressure-temperature-time (P-T-t) information from metasedimentary units deposited in proposed convergent settings from Archaean, Proterozoic and Phanerozoic terrains to characterize the evolution of minimum estimates of burial rate (km.Ma^−1^) as a function of the age of the rocks. The demonstrated decrease in burial rate correlates positively with a progressive decrease in the production of juvenile felsic crust in the Archaean and Proterozoic. Burial rates are also more diverse in the Archaean than in modern times. We interpret these features to reflect a progressive decrease in the diversity of tectonic processes from Archaean to present, coupled with the emergence of the uniquely Phanerozoic modern-style collision.

The volume of continental crust represents a balance between the addition of mantle-derived, “juvenile” material to the crust, and the recycling of crust into the mantle^1^ and it has changed through geological time. Several studies have attempted to model this evolution to best fit with the geological record^2^,^3^,^4^. Recently, based on Hf, U-Pb and O isotopic ratios in the zircon record, Dhuime *et al*.^5^ argued for a two-stage evolution of crustal volume as a function of time, with a period of rapid net crustal growth (3 km^3^.yr^−1^) with production of ~65% of the current continental mass prior to 3.0 Ga; followed by a decrease of net crustal growth rate to ~0.8 km^3^.yr^−1^ as a consequence of an increase in the recycling of the crust into the mantle (Fig. 1A). In today’s Earth, both juvenile growth and recycling are primarily a feature of convergent plate boundaries, especially subduction zones, and as a result the ca. 3.0 Ga transition is regarded by Dhuime *et al*.^5^ to reflect major changes in the nature of convergent plate boundaries, interpreted as the beginning of modern plate tectonics. Following its formation, in convergent settings, the crustal material undergoes reworking via erosion of the continental/arc landmass, burial of the peri-orogenic sedimentary basins, metamorphism and possible partial melting to produce crustally derived felsic magmas^6^. Thus, many if not most of the present-day crustal rocks record multiple episodes of reworking, as demonstrated by the global distribution of Hf isotope data^7^,^8^. Reworking is also a significant process that is responsible for, or accompanies, the stabilization of large continental domains (cratonization) and the redistribution and transport of heat producing elements within the crust via partial melting of lower crustal units^9^,^10^. Consequently, as the continental mass becomes more stable, reworking/cratonization in accretionary settings ultimately favors net crustal growth.

The chemical (bulk rock Na_2_O/K_2_O ratio) and isotopic signature (∂^18^O = ^18^O/^16^O in magmatic zircons) (Fig. 1B) of granitoid rocks, taken as a proxy for the bulk crustal composition, display important changes in the time interval 2.5 to 3.0 Ga^11^. The ∂^18^O values of magmatic zircons which record concordant U/Pb ages, show a marked increase in the late Archaean, from values close to the mantle ratio of 5.3 ± 0.3%, to much higher values, characteristic of rocks having experienced atmospheric weathering, or their derivatives^12^. Hence, the augmentation of ∂^18^O in igneous zircons after 3.0 Ga indicates that the sources of granite magmas become dominated by crustal material that has experienced weathering and clay formation^13^,^14^, and thus points to an increased reworking of existing crustal masses. This change is accompanied by a decrease in the Na_2_O/K_2_O ratio in the composition of both sediments and felsic igneous rocks^11^,^15^. This evolution reflects two superposed processes: (1) the increasing role of deep crustal melting of thickened continental sources^16^, recorded by the emergence of S-type granitoids (i.e., reworking); and (2) the change in composition of felsic additions to the continental crust from exclusively sodic (the Archaean TTG suite) to include potassic and high-Mg granitoids^17^,^18^. Consequently, the 2.5–3 Ga period sees changes in both the nature of juvenile magmatic material (nature, and presumably rates, of crustal growth) and an increase in the intensity of reworking mechanisms. The combination of both effects might be interpreted to reflect the switch from a situation with abundant crustal accretion with (presumably) low recycling rates, to a situation with enhanced recycling rates, but also reduction in the variety of mechanisms by which the crust is recycled.

In the present Earth, accretion of juvenile material, recycling of crust to the mantle, and crustal reworking by anatexis, all occur chiefly in convergent plate boundaries, and in particular in accretionary orogens, which correspond to the compaction of sedimentary wedges and island arcs onto preexisting continental mass. This may be followed by collisional orogens, which characterize the termination of the Wilson cycle with the merging of continental blocks^19^. Accretionary, as well as collisional orogens, are sites dominated by the thickening and burial of rocks which were once at surface. In examples where sedimentary deposits are buried deep enough, this might favor recycling and promotes reworking. Importantly, accretionary orogens produce transient yet substantial thermal anomalies with colder than average apparent geothermal gradients associated with accretionary prisms and the subducted slabs, and hotter than average apparent geothermal gradients produced in back-arc settings. Therefore, the aim of this contribution is to characterize the thermal patterns (geothermal gradients as well as burial rates) in convergent plate boundaries, including proposed Archaean equivalents, and to correlate them with information on crustal accretion, recycling and reworking. In order to address this question, we focus on the P-T-t information contained in the metasedimentary units related to accretionary and collisional orogenic settings. The combination of the metamorphic and detrital information allows the determination of minimum rates of tectonic burial that were used as a proxy to measure the rates at which supracrustal material is heated and transported to recycling/reworking sites. The careful investigation of burial rate in convergent settings in the past geological record might provide insights into the evolution of the mechanisms that shaped the face of Earth for the past 4.0 Ga. We contrast burial rates of Phanerozoic sediments with relatively well-defined geodynamic settings, with a set of Precambrian rocks from different orogenic belts, where details of the geodynamic processes are less well constrained.

## Methods

**Figure 2:**
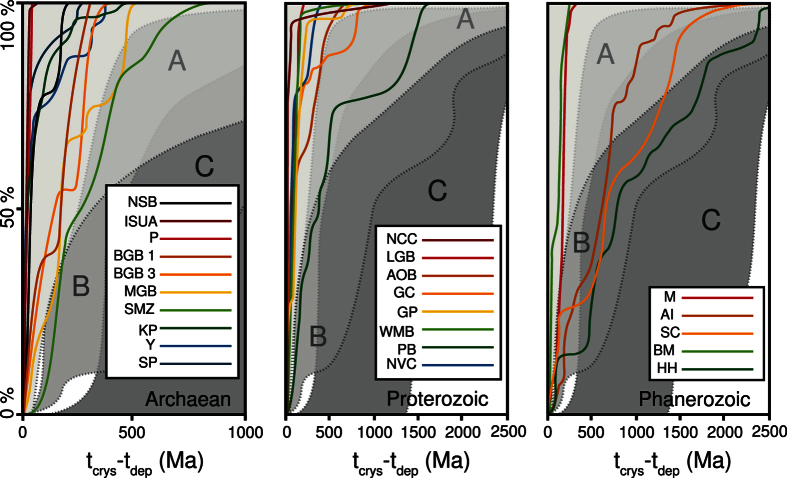
Detrital zircon record in the studied metasedimentary units. Cumulative probability density plot represented as a function of t_crys_-t_dep_ (t_crys_: ages of the inherited zircon in the sedimentary sequence; t_dep_: age of the deposit of the sedimentary sequence). As the population of inherited zircon varies with the depositional environment (i,e. tectonic setting), this technique allows discrimination between three main geodynamic settings of sedimentation: (**A**) Convergent (island arc, fore arc, back arc, trench), (**B**) Collisional and (**C**) Extensional basins (after Cawood *et al*.^20^). We used this method to constrain the database and only investigated convergent and collisional settings, excluding extensional environments from the compilation. This information alone cannot account for the type of mechanism driving plate motion and terrane accretions. Abbreviations in Tables 1, 2, 3.

**Figure 3:**
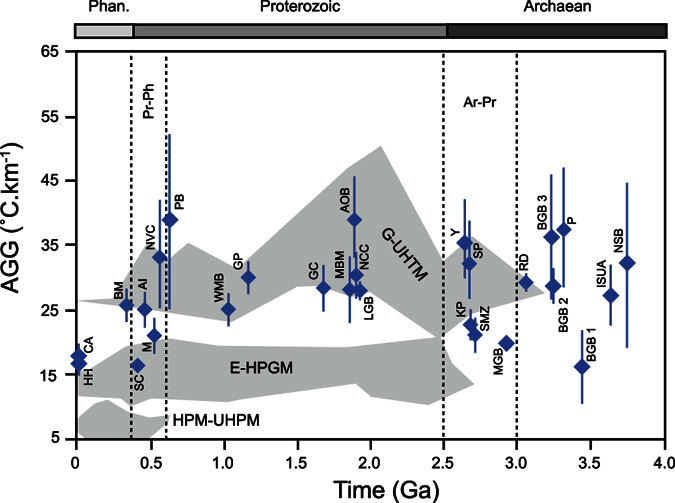
Evolution of the apparent geothermal gradient (AGG) for the last 4.0 Ga. Average apparent geothermal gradient values: Phanerozoic = 20.68** **°C.km^−1^; Proterozoic = 31.46** **°C.km^−1^; Archaean = 28.58** **°C.km^−1^ (apparent geothermal gradient standard deviation σ: Phanerozoic, 4.17; Proterozoic, 5.01; Archaean, 7.29). For the Archaean and Proterozoic, the average apparent geothermal gradient is consistent with a melt-enhanced geotherm^45^ as the peak metamorphic conditions exceed the fluid-absent solidus for clastic metasediments and granites. Therefore, the apparent geothermal gradient alone cannot be used to discriminate different types of orogenic settings. The horizontal time scale represents the recorded age of metamorphism. The grey areas represent the variation in recorded apparent geothermal gradient values as a function of metamorphism from the compilation by Brown^22^ (G-UHTM: granulite – ultrahigh temperature metamorphism; E-HPGM: ecolgite – high pressure granulite metamorphism; HPM-UHPM: high pressure – ultrahigh pressure metamorphism). Abbreviations in Tables 1, 2, 3.

Metamorphic pressure-temperature estimates combined with geochronological studies provide a unique opportunity to follow the evolution of a rock unit in depth-time space. Metasedimentary rocks contain both detrital and metamorphic zircon crystals that allow constraints to be placed on the minimum time between deposition of the sediment and peak metamorphism, thereby constraining the burial rate. Apparent metamorphic gradient was determined by the ratio T_p_/D (°C.km^−1^), with T_p_ representing the temperature at peak pressure and D: depth at peak pressure; and burial rate was determined by the ratio D/∂t (km.Ma^−1^), with ∂t representing the time gap between maximum sedimentary depositional age and peak metamorphic age. The samples chosen for this study display information in their detrital zircon record indicating that the sediments were deposited in what is typical for modern convergent and/or collisional settings^20^ (Fig. 2). As the sedimentary successions are susceptible to have undergone different metamorphic events during their history, we avoided polymetamorphic terrains. However, this becomes more difficult as the rocks get older. Hence, the samples from the Archaean Eon have been carefully selected and only rocks in which different metamorphic episodes can be clearly distinguished were included in the dataset (Fig. 3). The samples were separated into three age groups: Archaean (2.5–4 Ga), Proterozoic (0.54–2.5 Ga) and Phanerozoic (0–0.54 Ga). The full compilation of geochronological data, metamorphic conditions, apparent geothermal gradients, burial rates and relevant references can be found in Tables 1, 2, 3.

## Results

### Origin of metasedimentary units

Cawood *et al*.^20^ argued that the detrital zircon record within sedimentary rocks can provide insights into the type of environment within which the sediment was deposited. The time gap between the estimated deposit age, the age of the detrital zircons, as well as the age distribution within the inherited detrital zircon population, permits the distinction between three different types of geodynamic environments: convergent setting, collisional setting and extensional setting (Fig. 2). The detrital zircon record in the metasedimentary rocks included in this study demonstrate the Precambrian rocks investigated, where tectonic setting is the subject of debate, display similar convergent or collisional zircon patterns to those formed in much younger sedimentary sequences where the geodynamic environment is clearly defined. In addition, the Proterozoic-Phanerozoic transition is marked by an increase in the proportion of rocks displaying a collisional orogenic signature (Fig. 2). The detrital zircon record combined with the range of metamorphic conditions are consistent with the metasedimentary rocks having a magmatic arc or back arc origin (high T) with in some cases an accretionary prism component (low T)^19^ (Fig. 3). Hence, the dominant convergent setting pattern in the Archaean might either be interpreted to be the result of early subduction related plate tectonic mechanisms or to be the result of crustal shortening without systematic subduction of oceanic lithosphere, in which lateral plate motion and terrane accretion are driven by mantle traction rather than slab pull^21^.

### Apparent metamorphic gradients

Brown^22^ compiled a large database of metamorphic conditions through time in all sorts of lithologies. Although our dataset is smaller (Tables 1, 2, 3), as it includes only metasedimentary samples for which age data are available, it does partially reproduce the main findings of Brown^22^. Archaean samples show a large range of apparent geothermal gradients (>15 to <40 C°.km^−1^) whereas Proterozoic average apparent geothermal gradients display higher and narrower values (>25 to 40 °C.km^−1^). In the Phanerozoic, the apparent geothermal gradients are lower than those identify in the other two eons and vary from >15 to 25 C°.km^−1^ (Fig. 3).

### Minimum burial rates

**Figure 4:**
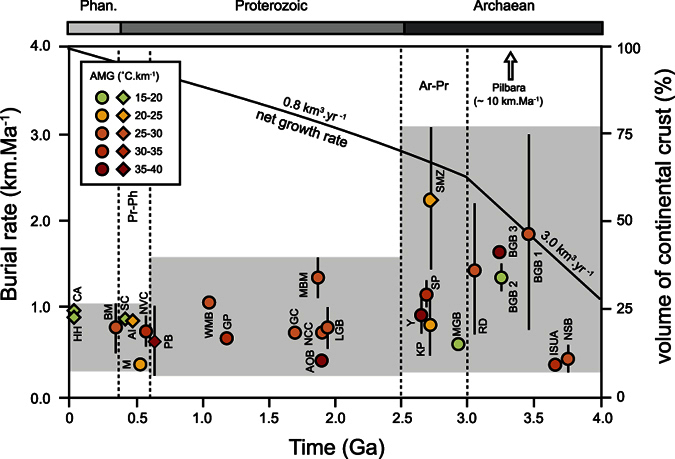
Compilation of the average burial rates recorded by different metasedimentary units over the past 4.0 Ga. Net growth rate curve by Dhuime *et al*.^5^. Circles represent proposed accretionary orogens and diamond represent proposed collisional orogens (after classification in Fig. 2). The grey envelope shows the range of burial rate values within the three different Eons. The Pilbara craton is indicated by the arrow. Ar-Pr: Archaean-Proterozoic, Pr-Ph: Proterozoic-Phanerozoic. Burial rate standard deviation σ: Archaean, 0.6; Proterozoic, 0.28; Phanerozoic, 0.24. Abbreviations in Tables 1, 2, 3, time scale: metamorphic ages.

The key new information in our database is related to the duration of burial processes (Fig. 4). The evolution of burial rates can be divided according to a three step sequence; (1) Archaean: 2.5–4.0 Ga., 0.29–3.06 km.Ma^−1^, (2) Proterozoic: 0.5–2.5 Ga., 0.21–1.34 km.Ma^−1^, (3) Phanerozoic: 0.0–0.5 Ga., 0.31–1.03 km.Ma^−1^. The Pilbara burial rate^21^ is very high (~10 km.Ma^−1^) – in fact it is unique, and may suggest that the Pilbara processes are unique, even within the Archaean Eon (see discussion below). Hence, it might be considered that average Archaean burial rate values vary between ~0.30 and ~3.0 km.Ma^−1^. The most important information drawn from this compilation is that the low burial rate characterizing the Phanerozoic (0.3–1 km.Ma^−1^) appears to have existed since 3.7 Ga as a background signal that seems to become more important over time (Fig. 4). Consequently, as the low rate appears to be a constant, the parameter that changes with time is the maximum rate of burial (Fig. 4). The apparent decrease in maximum recorded burial rate could correspond to important transitions already identified in the geological record and correlates with the proposed decrease in the net crustal growth speed at the Archaean-Proterozoic transition (2.5–3.0 Ga)^5^,^6^ (Fig. 4). The Proterozoic-Phanerozoic (0.5 Ga) transition is less well marked but seems to correlate with the emergence of a modern collisional orogeny pattern in the detrital zircon record.

## Discussion

### Significance of burial rates

Here we discuss the significance of burial rates and what they might be a proxy for. Numerical models of the tectonic settings within which crustal shortening occurs, such as the ones this study focuses on, propose that for rocks within a subduction/collision context, burial rates are equivalent to shortening rates^23^, i.e. 10–200 km.Ma^−1^. On the other hand, Thompson *et al*.^24^ showed that for a range of different geotherms, variable sizes of orogenic belt and degree of additional mantle heat flux, crustal thickening rates vary from 0.1 to 4.5 km.Ma^−1^. Our observations are in better accordance with the latter. Consequently, it is likely that burial rates only represent the speed at which supracrustal material is brought to recycling/reworking sites, under different geothermal conditions. Understanding the source of the discrepancy that appears to exist between observed and some calculated rates would require new numerical investigations. The use of our dataset might possibly help to better constrain future numerical modeling of geodynamic processes.

There is a decrease in maximum apparent burial rate at the end of the Archaean, from 3.06 km.Ma^−1^ to 1.34 km.Ma^−1^ (Fig. 4). In the Archaean, Pilbara burial rate appears to be an anomalous value as it records a very high burial rate which appears to be unique in the global geological record. Pilbara is also proposed to represent the most convincing example of “vertical tectonics” (sagduction) in the Archaean^25^,^26^. This process could have been present as a crustal recycling mechanism in the hotter Archaean Earth^27^,^28^ before the onset of global plate tectonics at 3.0 Ga^5^. Based on the present dataset, it could even be argued that the East Pilbara craton is the only example of such a process. In any case, it remains an example of a uniquely Archaean (pre-3.0 Ga) situation.

The evolution of the time gap (∂t) between deposition of the sediment and peak metamorphic age, which mainly influences the variation of burial rates within the three eons, seems to be related to the variation of the apparent geothermal gradient with time (Fig. S1). In the Archaean, the greater range of apparent geothermal gradient values seems to correlate with the large range of ∂t values (1–90 Ma). In the Phaneroizoic and the Proterozoic, ∂t values are less scattered (23–53 Ma) and correspond to apparent geothermal gradient values of >15 to 25 C°.km^−1^ and >25 to 40 °C.km^−1^ respectively (Fig. 3). Consequently, the decrease in burial rates is likely to be linked to the evolution of Earth’s thermal regime. In the last ~600 Ma, slower and more homogeneous burial rates might have been the consequence of ubiquitous cold subduction and low geothermal gradients. Hence, in the Precambrian, the important diversity in burial rates might highlight the presence of abundant and various accretionary settings as the consequence of a rheologicaly and thermally heterogeneous young lithosphere (i.e. different speeds of maturation process in different locations).

### Evolution of the continental crust

**Figure 5:**
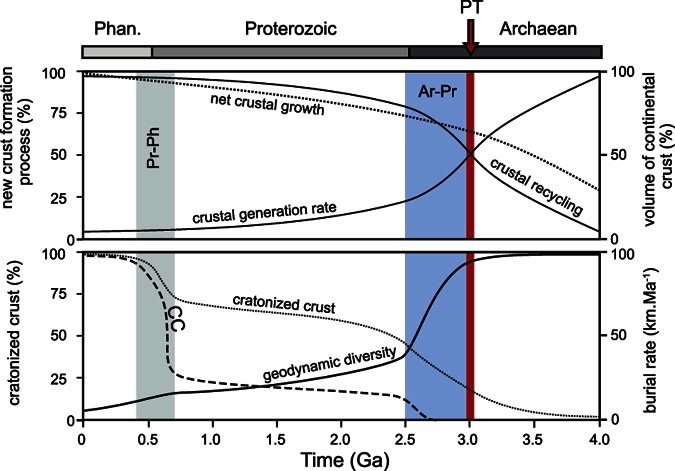
Possible evolution of crustal processes over the last 4.0 Ga. This speculative diagram illustrates change in the nature of the newly formed crust at the Archaean-Proterozoic (Ar-Pr) transition and the gradual decrease in diversity of geodynamic processes at convergent margins as Earth aged. The Ar-Pr period sees the onset of modern plate tectonics (PT)^5^ and the first evidences of continental collision (CC)^46^. The Ar-Pr transition is period when cratonization (i.e. reworking) processes are triggered and lasts until the end of the Proterozoic. The preservation of CC pattern in convergent settings becomes more marked at the Proterozoic-Phanerozoic (Pr-Ph) transition, possibly illustrating a state of full maturation of the crust.

Here we use changes in the range of orogenic burial rates, combined with changes in the chemical and isotopic composition of clastic sediments and granitoid rocks of the continental crust, to constrain the evolution of supracrustal units. We suggest this set of information might give possible insights into the important geodynamic changes over the last 4.0 Ga. Figure 5 graphically summarizes the possible scenario mentioned below for the evolution of crustal processes.

The pre-3.0 Ga period saw a large diversity of burial rates and apparent geothermal gradients (Fig. 4). High burial rates (e.g. Eastern Ghats Mobile Belt^29^,^30^, Barberton greenstone Belt^31^,^32^,^33^), possible super high burial rate (e.g. Pilbara^25^) (Fig. 4) and poor detrital zircon records in some early convergent settings (Fig. 2) indicate fast reintroduction (i.e. short time gap between sedimentary deposit and metamorphic event) of the near accretionary zone supracrustal material back to the mantle or the lower crust. This is in good agreement with the constant ∂^18^O in magmatic zircons (5–7% VSMOW) and high Na_2_O/K_2_O ratio (1.5–2.5)^12^, which highlight the low flux of weathered material into sedimentary deposit settings due to short time residency and low erosion profile^34^. However, rocks recording lower burial rates and richer detrital zircon records do exist from this period (e.g. Nuvvuagittuq Supracrustal Belt^35^,^36^; Murchison greenstone belt^37^,^38^; Isua Belt^39^,^40^) (Figs 3 and 4). The pre-3.0 Ga period crustal mechanisms were dominated by juvenile crust production. Important magmatic activity combined with a large range of burial rates might be interpreted to reflect diversity in the number, the length, the geometry and shortening speed of the convergent settings^1^. Hence, the pre-3.0 Ga period seems to be characterized by a large variety of geodynamic mechanisms that might vary from pure lateral shortening^33^,^41^ to more pronounced vertical displacements and lithospheric delamination processes^23^,^24^,^25^.

The Archaean-Proterozoic transition (2.5–3.0 Ga) was a period of massive modification of crustal processes. Recycling and reworking mechanisms progressively became dominant in convergent settings^42^ as more supracrustal material was reintroduced to lower crust or lithospheric mantle depth. This is demonstrated by the increase of ∂^18^O towards higher values (7.5–11% VSMOW) and the increase in the potassium content of felsic magmas^12^. This correlates also with the onset of modern plate tectonic mechanisms^5^, the emergence of the first potential signs of continental collision mechanisms^43^ (Figs 3 and 4) and the building of an emerged thicker landmass^34^. These events triggered an increasing discharge of detrital material in the convergent zone and more cannibalistic recycling/reworking^12^,^15^,^16^. The maximum tectonic burial rate declines as the net crustal growth rate decreases by a factor of 3. The Archaean-Proterozoic transition is interpreted as a period when cratonization of the lithosphere is triggered by the burial of a large amount of differentiated surpracrustal material in convergent zones. This transition period might correspond to the moment when modern plate tectonics emerged to be the more efficient mechanism by which recycling of the increasing sedimentary discharge is accommodated. As a consequence, the diversity in tectonic settings that might have been existed in the pre-3 Ga period rapidly decreased in favor subduction and continental collision.

The Proterozoic is interpreted to be a period of long-lived orogens (up to 700 Ma)^44^, which may have potentially enhanced crustal recycling. The range of tectonic burial rates in lateral accretion settings appears to decrease slightly from 2.5 ga to 0.5 Ga (maximum values from 1.34 km.Ma^−1^ to 1.01 km.Ma^−1^)(Fig. 4). The important recycling of material enriched in heat production elements (i.e. detrital and felsic products rich in K, U, Pb and Th) changed the thermal structure of the crust^13^. The Proterozoic is characterized by a homogeneous high apparent geothermal gradient (31.46 °C.km^−1^) (Fig. 3). Chardon *et al*.^45^ showed that this geothermal signature can be related to a period dominated by hot orogens These were characterized by distributed deformation in long lasting convergence, horizontal and vertical advection and high topography, as well as mixed orogens, characterized by accretion of magmatic arcs and juvenile materials with deformation localized in a rigid upper mantle. From this perspective, the Proterozoic Eon represents a period of crustal maturation and assimilation of the recycled and reworked products that were progressively introduced into the system since 2.5–3.0 Ga.

After several cycles of formation and break-up of supercontinents^6^, the range of burial rates became less scattered (maximum value ~1 km.Ma^−1^) as a consequence of the progressive cratonization of the continental landmass. Evidence for modern continental collision is more pronounced after *ca.* 0.5 Ga. However, continental collision was effective since the Paleoproterozoic as indicates by Nuna and Kenorland supercontinents formation^46^. The sudden preservation of continental collision signature could illustrate a state of near-full maturation (i.e. full cratonization) of the continental lithosphere. Results drawn from this study are in agreement with findings by Scholl & von Huene^1^ who suggested that almost the entire mass of continental crustal material might have undergone recycling and reworking mechanisms over the past 4.0 Ga. Consequently, the decrease in diversity in burial rate in convergent zones seems to correlate positively with the progressive decrease in net crustal growth and the increase of the occurrence of reworked and recycled material. Thus, after 4.0 Ga of Earth history, relatively slow rates of tectonic burial appear to have been selected at the expense of higher speed processes to efficiently accommodate the progressive cratonization of the lithosphere, the global cooling of the Earth^27^,^28^ and maintain the Earth geodynamics in a steady state via the generalization of modern continental collision settings.

## Conclusion

Metamorphic, geochronological, chemical and isotopic information contained in metasedimentary and felsic rocks of current and past lateral shortening sites indicate the evolution of the mechanisms that shape the face of the Earth for the past 4.0 Ga are the direct expression of the competition between new crustal formation and assimilation of the buried supracrustal material. Therefore, rather than to debate the existence/non-existence of lateral tectonics, it seems more appropriate to address the issue of the evolution of tectonic regimes (i.e. the classic “Archaean” type vs. “modern” type consensus), from the point of view of lithospheric processes efficiency. In the Archaean Eon, fast processes indicate low residency time, hence less material in convergent settings and poor assimilation. In the Proterozoic and Phanerozoic Eons, slower burial rates show higher residency time, therefore better assimilation of the supracrustal material in recycling and reworking sites.

The modern geodynamic regime is the result of a 4.0 Ga long history that saw convergent plate tectonics progressively became the dominant mechanisms and the emergence of strongly marked modern collisional orogenic signature at the end of the Precambrian. The current dataset is not exhaustive. A systematic metamorphic and geochronological investigation of Precambrian terrains would help to strength this hypothesis. More work is needed to properly understand this evolution in regard of the global decrease of Earth thermal regime over time. Nevertheless our work provides material to better constrain numerical models and new hypotheses for a unified theory for the formation and the evolution of the lithosphere.

## Additional Information

**How to cite this article**: Nicoli, G. *et al*. Diversity of burial rates in convergent settings decreased as Earth aged. *Sci. Rep.* **6**, 26359; doi: 10.1038/srep26359 (2016).

## Supplementary Material

Supplementary Information

## Figures and Tables

**Figure 1:**
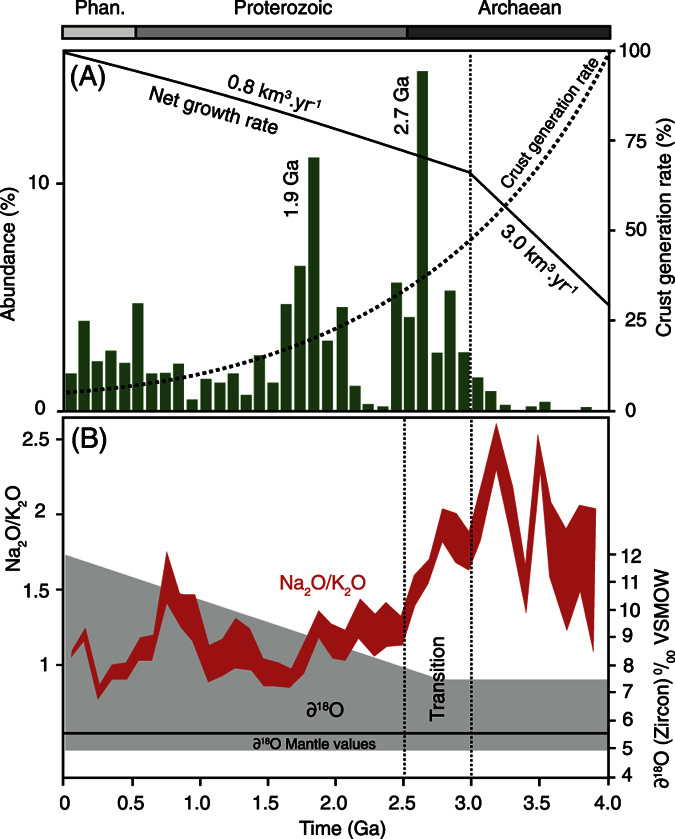
Records of changing modes of crustal growth, recycling and reworking through time (zircon ages). (**A**) The black curve shows the two stages model for net continental growth using Hf, U-Pb and O isotopic values from the zircon magmatic record^5^. The dashed curve represents the model in which new juvenile crust formation decreases over time^6^. The peak ages in the detrital zircon record at 2.7 Ga and 1.9 Ga have been interpreted to reflect episodes of juvenile crust formation, yet are more likely artifacts produced by preferential preservations of continental crust during supercontinent cycle and do not account for the real proportion of newly formed crust generated during these periods^6^. (**B**) Evolution of the ratio of Na_2_O/K_2_O in the felsic crustal components^11^ and evolution of the ∂^18^O value in the magmatic zircon^12^.

**Table 1:** Compilation of metamorphic and geochronological data for the Archaean Eon (2.5–4 Ga).

Archaean		Deposit (Ma)	Metamorphism (Ma)	T_p_ (°C)	P (kbar)	Grade	AMG (°C.km^−1^)	Burial rate (km.Ma^−1^)	References
Northeastern Superior Province									
NSB	Nuvvuagittuq Supracrustal Belt	3780 ± 22	3738 ± 25	615 ± 35	5 ± 2	Am	32.21 ± 13.4	0.43 ± 0.1	35,36
Isua Belt, Greenland									
ISUA	Isua	3699 ± 12	3630 ± 30	610	6 ± 1	Am	27.45 ± 4.6	0.32 ± 0.1	39,40
Pilbara, Australia									
P	Eastern Pilbara Province	3314 ± 3	3311 ± 5	700 ± 50	5.5 ± 1.5	Gr	37.84 ± 9.7	10 ± 4.8	25
Kaapvaal Craton, Southern Africa									
BGB 1	Barberton GB	3453 ± 6	3436 ± 18	535 ± 15	5 ± 1	Am	28.88 ± 2.7	1.86 ± 1.1	31,33
BGB 2	Barberton GB	3260 ± 10*	3233 ± 17	575 ± 25	9.5 ± 1.5	Am	16.33 ± 5.8	1.34 ± 0.1	33,38
BGB 3	Barberton GB	3240 ± 4	3231 ± 5	640 ± 40	4.8 ± 1.0	Am	38.12 ± 7.8	1.65 ± 0.1	32
MGB	Murchison GB	2979 ± 12	2923 ± 11	625 ± 25	8.5 ± 0.5	Am	19.97 ± 1.4	0.56 ± 0.1	37,38
Eastern Ghats Mobile Belt, India									
RD	Rengali Domain	3087*	3057 ± 17	849 ± 31	7.8 ± 0.13	Gr	29.38 ± 1.2	1.41 ± 0.8	28,29
Limpopo Belt, southern Africa									
SMZ	Southern Margnal Zone	2733 ± 13	2713 ± 8	852 ± 8	11.1 ± 1.3	Gr	21.09 ± 2.4	2.26 ± 0.8	43
Fennoscandian Shield, Finland									
KP	Karelia Province	2740	2680 ± 30	825 ± 25	10.0 ± 1.0	Gr	22.57 ± 2.3	0.78 ± 0.3	47
Yilgarn, Australia									
Y	Eastern Gold Field	2658 ± 3	2640 ± 10	563 ± 14	4.2 ± 0.7	Am	36.18 ± 6.1	0.95 ± 0.2	48
Superior Province, Canada									
SP	Quetico	2694 ± 4	2677 ± 7	700 ± 70	6 ± 1	Gr	32.94 ± 6.1	1.16 ± 0.1	49

Database for the metasedimentary units including deposit age (Ma) of the supracrustal units, timing of peak metamorphism (Ma); AMG: Apparent Metamorphic Gradient (**°**C.km^−1^); Am: Amphibolite facies metamorphism, Gr: Granulite facies metamorphism; and burial rates (km.Ma^−1^); *data for which the inherited detrital zircon population was not available or not well constrained.

**Table 2:** Compilation of metamorphic and geochronological data for the Proterozoic Eon (0.54**–**2.5 Ga).

Proterozoic		Deposit (Ma)	Metamorphism (Ma)	T_p_ (°C)	P (kbar)	Grade	AMG (°C.km^−1^)	Burial rate (km.Ma^−1^)	References
China Craton									
NCC	North China Craton	1970 ± 24	1919 ± 10	975	9.5 ± 0.5	Gr	27.79 ± 1.5	0.76 ± 0.2	50
Fennoscandian Shield									
LGB	Lapland Granulitic belt	1940	1900 ± 12	825 ± 25	7.5 ± 1.0	Gr	30.36 ± 4.1	0.73 ± 0.1	51
Siberian Craton									
AOB	Angara Orogenic belt	1943 ± 13	1890 ± 10	756 ± 40	5.3 ± 0.8	Gr	39.66 ± 6.2	0.37 ± 0.1	52
Arnhem Province, N. Australia									
MBM	Melville Bay Metamorphics	1883 ± 9	1860 ± 9	840 ± 80	8.3 ± 1.3	Gr	28.43 ± 5.0	1.34 ± 0.2	53
Gawler Craton, Australia									
GC	Kimban Orogeny	1730 ± 10	1686 ± 8	825 ± 25	8 ± 1	Gr	28.39 ± 3.6	0.69 ± 0.1	54
Grenville Orogeny, N. America									
GP	Grenville Province	1213.5 ± 6.5	1175 ± 5	725 ± 15	6.5 ± 0.5	Gr	30.12 ± 2.4	0.63 ± 0.1	55,56
Pan African Orogeny, Antarctica									
WMB	Western Maud Belt	1072 ± 10	1040 ± 10	850	9 ± 1	Gr	25.5 ± 2.8	1.04 ± 0.1	57
Pan African (Brasiliano) Orogeny, Brazil									
PB	Pernambuco Belt, Brazil	665 ± 34	626 ± 15	650 ± 100	4.25 ± 1.25	Am	38.99 ± 13.0	0.61 ± 0.4	58
NVC	Nova Venécia Complex	606 ± 3	570 ± 4	831 ± 21	7.15 ± 1.85	Gr	33.85 ± 8.2	0.73 ± 0.2	59

Database for the metasedimentary units including deposit age (Ma) of the supracrustal units, timing of peak metamorphism (Ma); AMG: Apparent Metamorphic Gradient (**°**C.km^−1^); Am: Amphibolite facies metamorphism, Gr: Granulite facies metamorphism; and burial rates (km.Ma^−1^).

**Table 3:** Compilation of metamorphic and geochronological data for the Phanerozoic Eon (0.0**–**0.54 Ga).

Phanerozoic		Deposit (Ma)	Metamorphism (Ma)	T_p_ (°C)	P (kbar)	Grade	AMG (°C.km^−1^)	Burial rate (km.Ma^−1^)	References
Pan African orogeny, Madagascar									
M	Vohibory Series	640 ± 7	531 ± 7	775 ± 75	10.0 ± 1.0	Gr	21.34 ± 3.0	0.34 ± 0.1	60
The Arunta Inlier, Australia									
AI	Harts Range Metamorphic Core Complexe	510 ± 10	470 ± 10	825 ± 25	9 ± 1	Gr	25.14 ± 2.9	0.83 ± 0.1	61
Caledonian Orogeny, N.W. Europe									
SC	Scandinavian Caledonides	462 ± 2	423 ± 1.7	544 ± 16	9	Gr	16.32 ± 0.5	0.85 ± 0.1	62,63
Variscan Orogeny, W. Europe									
BM	Bohemian Massif	387 ± 14	341 ± 6	750 ± 50	7.5 ± 5	Gr	26.06 ± 2.6	0.77 ± 0.3	64,65
Himalayan Orogeny									
HH	Higher Himalaya	63 ± 2	21 ± 3	725 ± 75	11 ± 1	Am	17.55 ± 2.5	0.97 ± 0.1	66,67
Alps, W. Europe									
CA	Central Alps	50*	18.0 ± 0.3	560 ± 30	8.5 ± 0.5	Am	17.72 ± 1.4	0.98 ± 0.1	68

Database for the metasedimentary units including deposit age (Ma) of the supracrustal units, timing of peak metamorphism (Ma); AMG: Apparent Metamorphic Gradient (**°**C.km^−1^); Am: Amphibolite facies metamorphism, Gr: Granulite facies metamorphism; and burial rates (km.Ma^−1^); *data for which the inherited detrital zircon population was not available or not well constrained.
