# Supplementary material for: Diversity of burial rates in convergent settings decreased as Earth aged
Source: Sci Rep. 2016 May 24;6:26359. doi: 10.1038/srep26359 (PMC4877656; doi:10.1038/srep26359)
Supplement: Supplementary Information [file srep26359-s1.pdf]

Diversity of burial rates in convergent settings decreased as Earth aged – Supplementary information

Gautier Nicoli, Jean-François Moyen, Gary Stevens

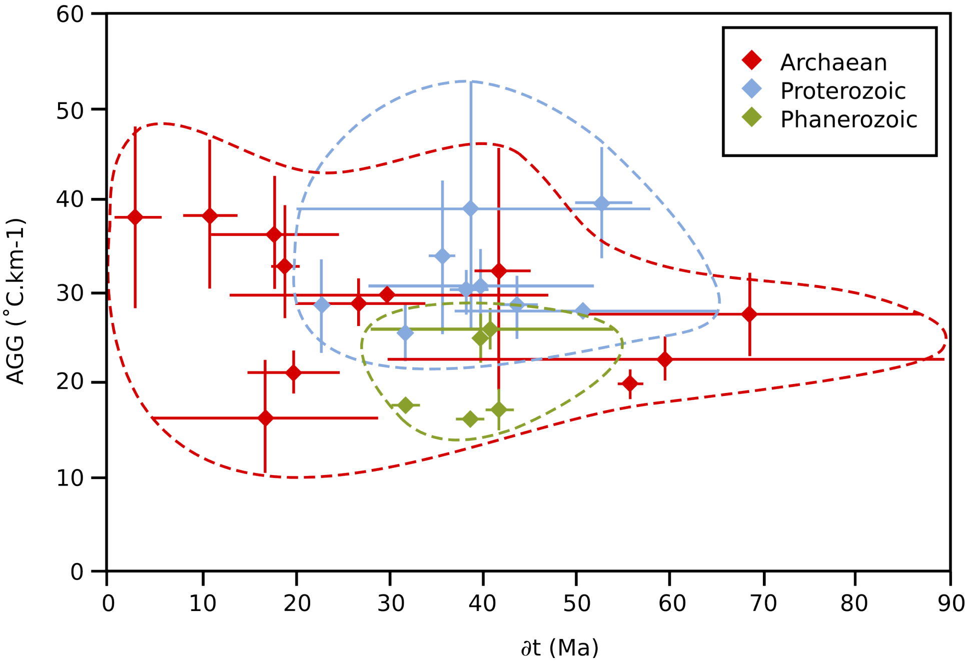

Figure 1S. Evolution of the apparent geothermal gradient (AGG) in metasedimentary belts as a function of the time gap between sedimentary deposit and peak metamorphic conditions (∂t). The dashed lines represent the set of possible AGG and ∂t for each eon. Despite greater error on the ∂t, the Archaean data cover a larger range of ∂t values (>0 to < 90 Ma) than Phanerozoic and Proterozoic data (30 to 55 Ma and 20 to 65 Ma respectively).
